# Development of Ferromagnetic Materials Containing Co_2_P, Fe_2_P Phases from Organometallic Dendrimers Precursors

**DOI:** 10.3390/molecules26216732

**Published:** 2021-11-06

**Authors:** Alaa S. Abd-El-Aziz, Maysun R. Benaaisha, Mohammed S. M. Abdelbaky, David Martinez-Blanco, Santiago García-Granda, Amani A. Abdelghani, Laila H. Abdel-Rahman, Rabin Bissessur

**Affiliations:** 1 Department of Chemistry, University of Prince Edward Island, 550 University Avenue, Charlottetown, PE C1A 4P3, Canada; mbenaaisha@upei.ca (M.R.B.); aabdelghani@upei.ca (A.A.A.); rabissessur@upei.ca (R.B.); 2 Department of Physical and Analytical Chemistry, University of Oviedo-CINN, 33006 Oviedo, Spain; saidmohammed.uo@uniovi.es (M.S.M.A.); sgg@uniovi.es (S.G.-G.); 3 Scientific and Technical Services, University of Oviedo-CINN, 33006 Oviedo, Spain; martinezbdavid@uniovi.es; 4 Chemistry Department, Faculty of Science, Sohag University, Sohag 82524, Egypt; Laila.abdelrahman@science.sohag.edu.eg

**Keywords:** cobalt-containing organoiron dendrimer, transition metal phosphides, ferromagnetic materials

## Abstract

The development of synthesis methods to access advanced materials, such as magnetic materials that combine multimetallic phosphide phases, remains a worthy research challenge. The most widely used strategies for the synthesis of magnetic transition metal phosphides (TMPs) are organometallic approaches. In this study, Fe-containing homometallic dendrimers and Fe/Co-containing heterometallic dendrimers were used to synthesize magnetic materials containing multimetallic phosphide phases. The crystalline nature of the nearly aggregated particles was indicated for both designed magnetic samples. In contrast to heterometallic samples, homometallic samples showed dendritic effects on their magnetic properties. Specifically, saturation magnetization (*M_s_*) and coercivity (*H_c_*) decrease as dendritic generation increases. Incorporating cobalt into the homometallic dendrimers to prepare the heterometallic dendrimers markedly increases the magnetic properties of the magnetic materials from 60 to 75 emu/g. Ferromagnetism in homometallic and heterometallic particles shows different responses to temperature changes. For example, heterometallic samples were less sensitive to temperature changes due to the presence of Co_2_P in contrast to the homometallic ones, which show an abrupt change in their slopes at a temperature close to 209 K, which appears to be related to the Fe_2_P ratios. This study presents dendrimers as a new type of precursor for the assembly of magnetic materials containing a mixture of iron- and cobalt-phosphides phases with tunable magnetism, and provides an opportunity to understand magnetism in such materials.

## 1. Introduction

Magnetic materials have recently attracted significant research interest, as they often show novel and improved properties over their bulk counterparts [1,2,3]. It is of scientific and practical significance in many fields ranging from advanced technology to biomedical applications [4,5,6,7,8]. Indeed, the synthesis of new magnetic materials remains an inspiring goal for many research groups [9]. Among the various magnetic materials applied in science, industry, and technology are transition metal phosphide (TMP) complexes, such as Fe_2_P, FeP, Co_2_P, CoP, Ni_3_P, Ni_2_P [10,11,12]. These materials are compounds between phosphorous and transition metals that combine physical properties, such as the hardness and strength of ceramics, with electronic and magnetic properties similar to metals [13]. Lately, the synthesis of magnetic TMP structures has become a promising approach to explore magnetic properties due to the formation of unusual magnetic fields, generation of quantum effects, and other phenomena [14]. In addition to their magnetic properties, the development of TMP complexes remains an attractive goal for researchers due to their high catalytic activity, such as a photocatalytic cocatalyst for hydrogen evolution reactions (HER), and hydrodenitrogenation (HDN) and hydrodesulfurization (HDS) [15,16,17]. Depending on the composition of the TMPs, two main classes have been recognized, phosphorus-rich and metal-rich phosphides [18]. However, metal-rich phosphides are the most exciting compositions between the two classes, as they possess metallic and ceramic properties, and exhibit high chemical and thermal stability [19]. The traditional strategy for producing metal phosphide is through the reaction between highly toxic phosphenes, PH_3_ or PCl_5_, and metals or metal salts [20]. For a less toxic procedure, P(SiMe_3_)_3_ was used as the P^3−^ ion source, which reacts with metal ions or metal carbonyl complexes at high temperatures [21,22]. Organometallic approaches have been extensively also used to develop transition metal phosphides [23,24,25]. Most of these researchers focused on synthesizing single TMPs hybrid materials [26,27,28,29,30] or on preparing a series of TMPs with the same metals [31]. All of these factors prompted us to explore the possibility of preparing ferromagnetic materials combining iron- and cobalt-phosphides through pyrolysis of hyperbranched macromolecules. This strategy is the most explored approach for synthesizing magnetic ceramic materials, which involves the high-temperature conversion of organometallic macromolecular precursors into magnetic materials [32,33].

Dendrimers are highly branched, monodisperse macromolecules with well-defined symmetric and globular structures [34,35,36]. Although much attention has been directed to dendritic structures in optics and biology, they have not been well explored in materials science [37]. In this regard, to increase the potential of dendrimers in this field, our group was interested in exploring organometallic dendrimers as a new class of precursors to design magnetic materials. The motivation for developing organometallic dendrimers is based on the idea that the metal-containing dendrimers may exhibit properties that are challenging to achieve or entirely inaccessible by using their pure organic relatives [38]. Here, we hypothesize that the presence of iron affects the magnetic properties of complexes derived from the organometallic dendrimers.

Moreover, the presence of iron stimulates dendrimers towards nucleophilic aromatic substitution reactions at the periphery [39], allowing peripheral functionalization of the dendrimers with magnetic species, further extending their potential for applications. Therefore, we consider these multifunctional organometallic dendrimers as a beneficial platform to establish their ability as precursors for ferromagnetic materials containing multimetallic phosphide phases. In this work, we exploited the sensitivity of organoiron dendrimers (**G1–D2 to G5–D9**) toward nucleophilic substitution reactions to synthesize iron- and cobalt-containing heterometallic dendrimers within dendritic branches. To understand the magnetic property of designed magnetic materials, five generations of homometallic and four generations of heterometallic dendrimers were synthesized, which, upon pyrolysis at 900 °C under N_2_ stream, produced magnetic samples containing a mixture of iron- and cobalt-phosphide phases in a good yield. At a fundamental level, this study presents a new method to develop ferromagnetic materials containing mixture phases of TMPs from organometallic dendrimers, allowing further exploration of the potential of macromolecules in the synthesis of magnetic materials, which may ultimately improve the design of precursors for magnetic materials containing multimetallic phosphide phases.

## 2. Results and Discussion

This article investigates the potential of using organometallic dendrimers as new precursors to design magnetic materials containing multimetallic phosphide phases. Here, four generations of a heterometallic dendrimer (**Co/Fe-dendrimer D3, Co/Fe-dendrimer D5, Co/Fe-dendrimer D7, and Co/Fe-dendrimer D9**) were designed, pyrolyzed, and studied for their magnetic properties. In addition, the magnetic properties of homometallic magnetic particles derived from homometallic dendrimers (**D3–D9**) were also examined.

### 2.1. Syntheses and Characterization of Homometallic Dendrimers

**Scheme 1:**
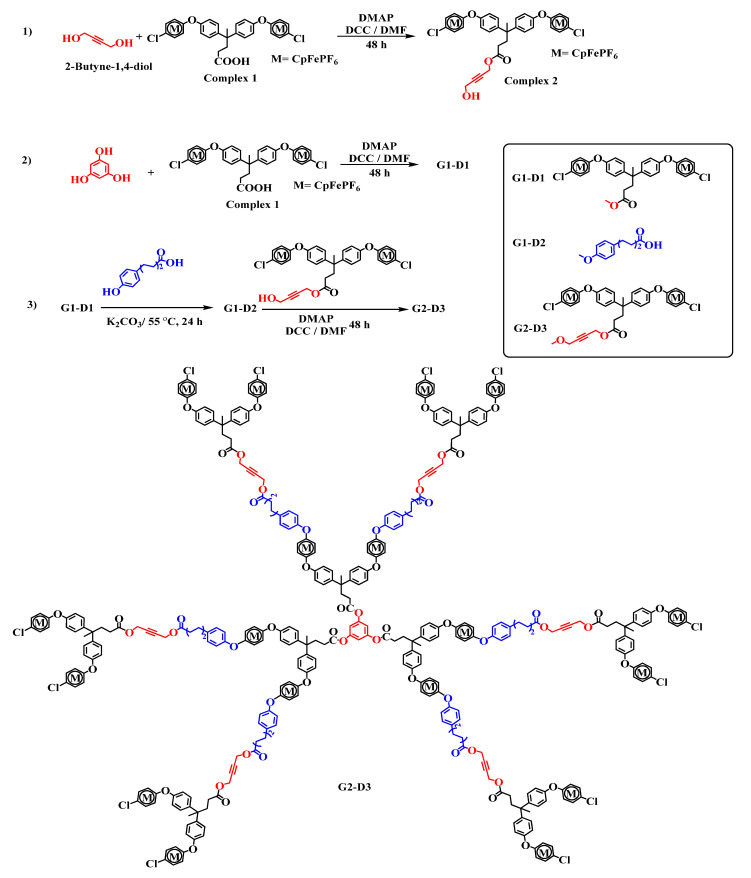
Schematic illustration of the synthesis of complex **2** and **HOMODENs G1–D1**, **G1–D2** and **G2–D3**.

**Scheme 2:**
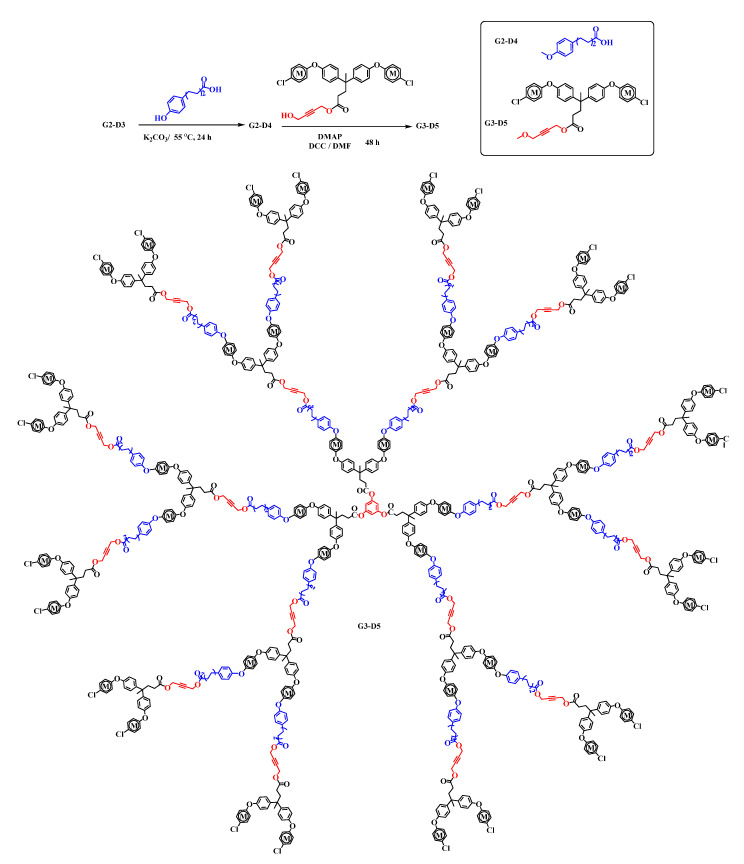
Schematic illustration of the synthesis of **HOMODENs G2–D4** and **G3–D5**.

**Scheme 3:**
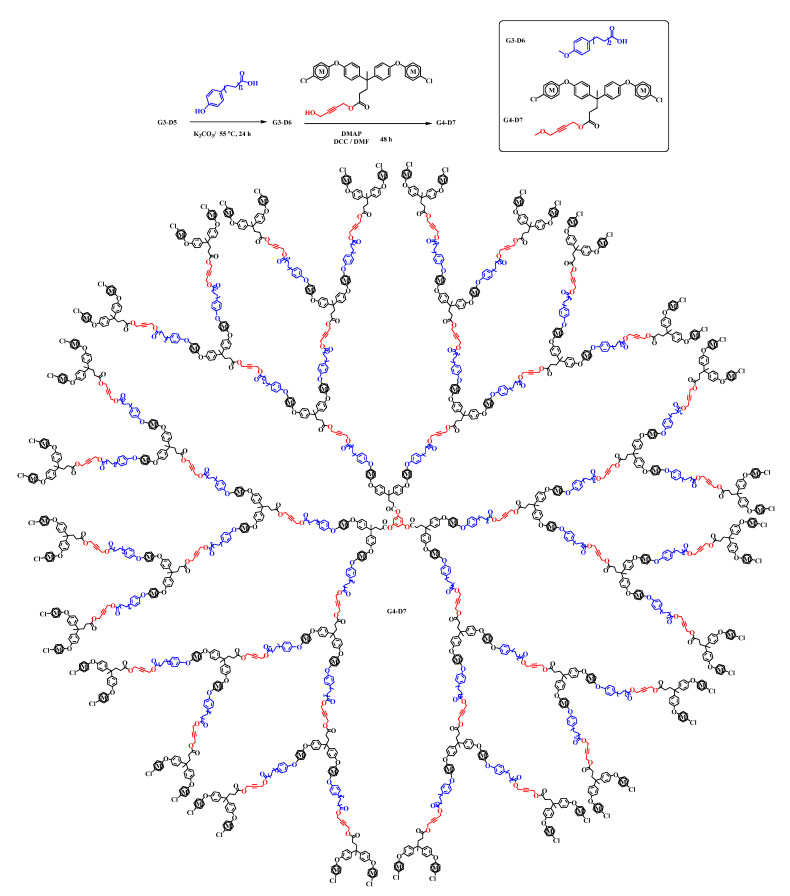
Schematic representation of the synthesis of **HOMODENs G3–D6** and **G4–D7**.

**Scheme 4:**
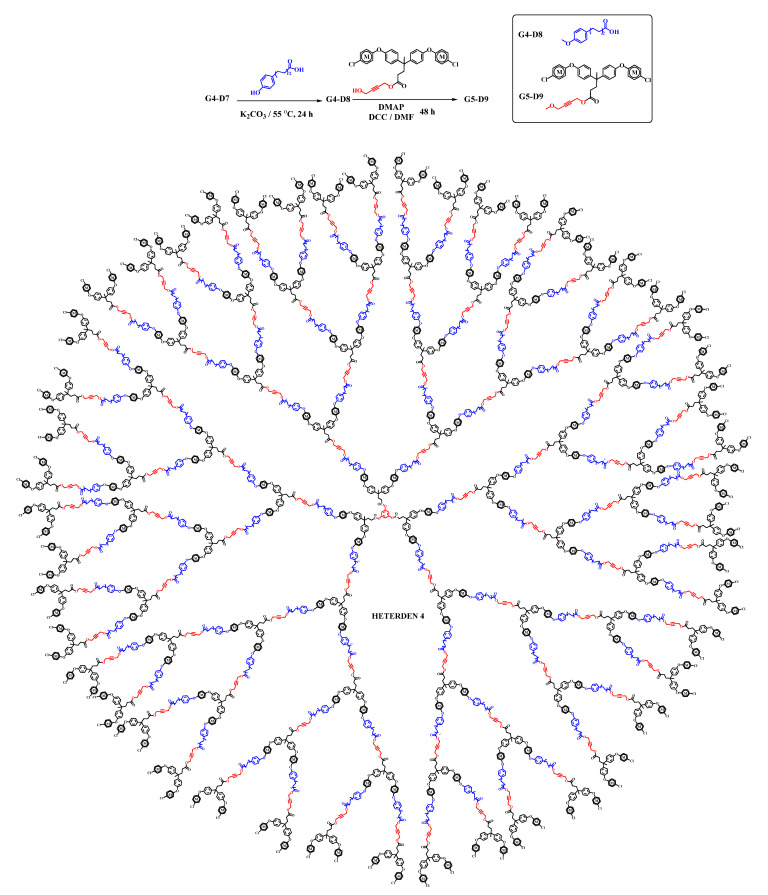
Schematic representation of the synthesis of **HOMODENs G4–D8** and **G5–D9**.

Five generations of homometallic dendrimers containing an alkyne group throughout their branches were synthesized using a divergent synthetic method, and a good yield was gained at around 87%. Steglech esterification reaction was used interchangeably with nucleophilic aromatic substitution reaction (S_N_Ar) to build dendrimer generations (Scheme 1, Scheme 2, Scheme 3 and Scheme 4). Dendrimers’ structures were confirmed by ^1^H, ^13^C NMR spectroscopy, IR, and element analysis. Also, the thermal stability was characterized using TGA.

^1^H and ^13^C NMR, FTIR, and elemental analysis were used to confirm the synthesized homometallic dendrimers. The shift in ^1^H NMR peaks of homometallic dendrimers supported their successful syntheses. For instance, terminal carboxylic group dendrimer **G1–D2** showed one signal at 6.23 ppm due to equivalent surrounding etheric oxygen groups, which refers to 24 protons in the six complexed aryl groups. Similarly, one peak was detected for these complexed protons in dendrimers **G2–D4**, **G3–D6**, and **G4–D8**. Moving to the terminal chloro-group dendrimer **G2–D3**, three distinct peaks appeared at 6.81, 6.41, and 6.23 ppm. The upfield peak at 6.23 ppm indicated complexed aryl groups attached to etheric oxygen groups. While the other two peaks indicated outer complexed-aryl groups attached to the peripheral chloro-end group. Also, ^1^H NMR spectra of dendrimers **G3–D5**, **G4–D7**, and **G5–D9** exhibited similar observation peaks for the complexed aryl group protons.

Moreover, two small peaks were observed around 4.72 and 4.11 ppm in complex **2**, which indicate CH_2_ protons from the alkyne moieties attached to the carboxylic- and alcohol-end groups, respectively. These peaks were observed as a small broad peak at 4.76 ppm in **G2–D3**, **G3–D5**, **G4–D7**, and **G5–D9**, which confirms the success of the esterification reaction. Additionally, in terminal carboxylic group dendrimers, one peak at 5.21 ppm corresponding to the Cps protons was observed. In the chloro-end group dendrimers, two peaks were found at 5.21 and 5.28 ppm corresponding to the inner and outer Cps protons. It is also important to state that both Cp peaks showed integration in agreement with the ratio of Cp protons in the periphery attached either to chloro-arenes or to those in the inner attached to etheric oxygen groups. The integration of the peaks indicates which peak represents the inner and which one represents the outer Cp groups. The disappearance of the OH peak of the carboxylic group in the ^1^H NMR spectrum of **G2–D3**, **G3–D5**, **G4–D7**, and **G5–D9** is another indication of a successful esterification reaction between **G1–D2**, **G2–D4**, **G3–D6**, and **G4–D8** and complex **2**. This peak resonated at 12.06 ppm in **G2–D3**, **G3–D5**, **G4–D7**, and **G5–D9** dendrimers, and due to the formation of the ester group, the peak has been omitted. This observation confirmed the successful formation of higher-generation dendrimers.

Successful synthesis of five generations of dendrimers was also confirmed by using ^13^C NMR spectroscopy. For instance, a carbonyl group with three signals at 175.48, 173. 33 and 171.23 ppm were detected in most of the homometallic dendrimers’ spectra. Although three carbonyl peaks were expected in the **G1–D1** and **G2–D3** of chloro-end group homometallic dendrimers, only two signals were recorded at 170.25 and 173.02 ppm. The peak at 170.25 ppm was attributed to overlapped peaks of the carbonyl carbons of the ester linkages of the first and second generations of chloro-end group dendrimers, and one at 173.02 ppm referred to the ester linkage of the core. Similarly, a single peak in the dendrimers **G1–D2** and **G2–D4** corresponding to the Cp carbons around 78.70 and 78.68 ppm was observed. Two distinct Cp carbon peaks around 80.21 and 78. 68 ppm appeared in the higher generation dendrimers due to the growth of these generations.

Furthermore, the complexed CH aromatic appeared between 87.97 and 73.57 ppm, while the uncomplexed CH aromatic occurred between 134.18 and 107.25 ppm, and the C≡C carbons were detected around 53.92 and 49.76 ppm. The ATR-FTIR absorption spectra of the homometallic dendrimer exhibited the presence and characteristic bands of hydroxyl, ester, and ether groups, around 3315, 1705, and 1223 cm^−1^, respectively. Elemental analysis has further confirmed the formation of the homometallic dendrimer, as outlined in the experimental section.

### 2.2. Synthesis of Heterometallic Dendrimers

**Scheme 5:**
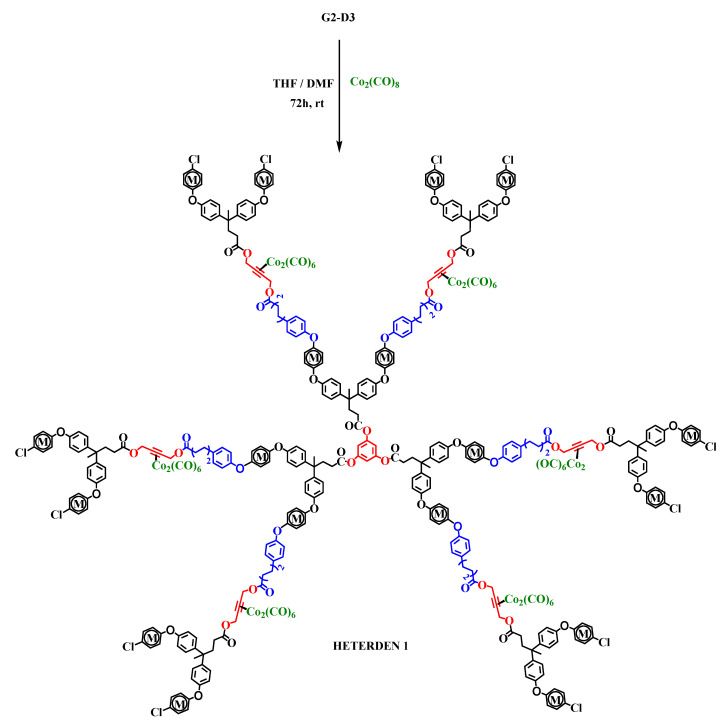
The synthesis of Co/Fe-dendrimer D3 (**HETERDEN 1**).

**Scheme 6:**
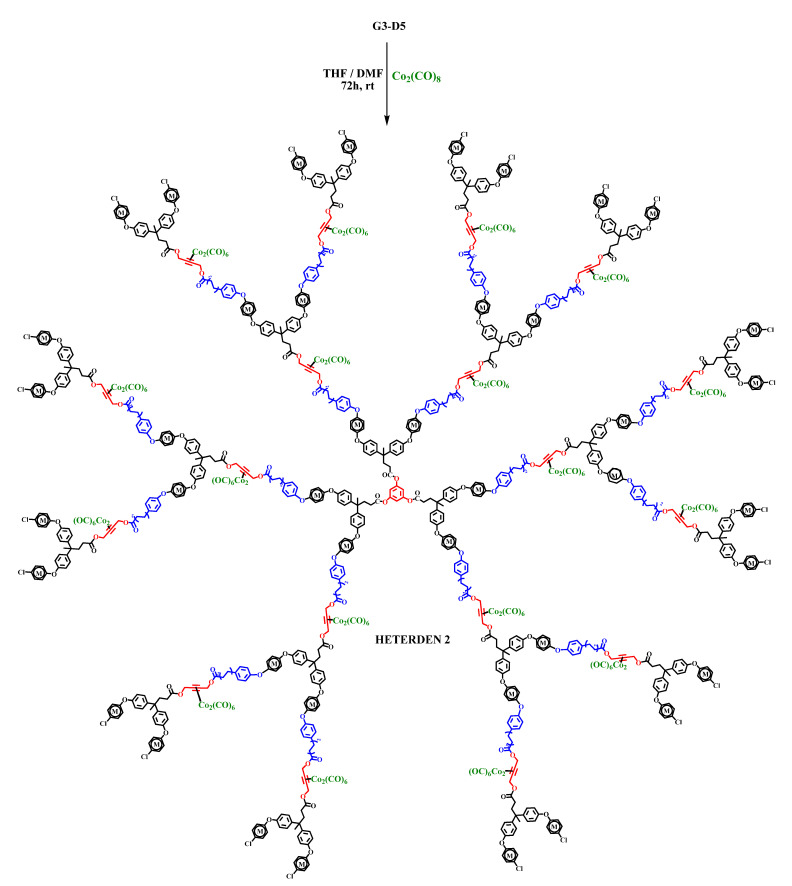
The synthesis of Co/Fe-dendrimer D5 (**HETERODEN2**).

**Scheme 7:**
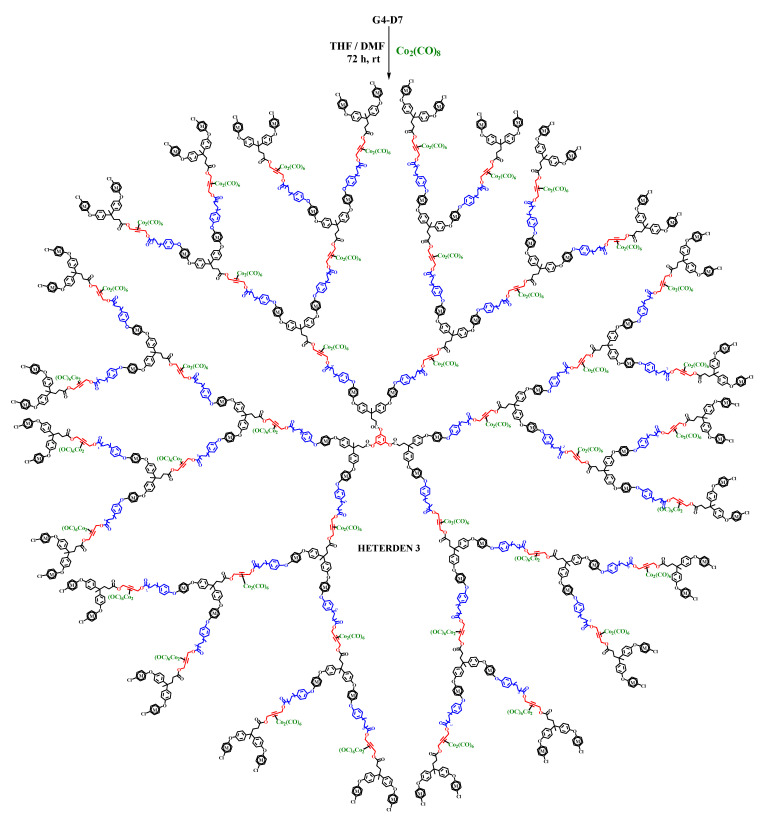
The synthesis of Co/Fe-dendrimer D7 (**HETERODEN3**).

**Scheme 8:**
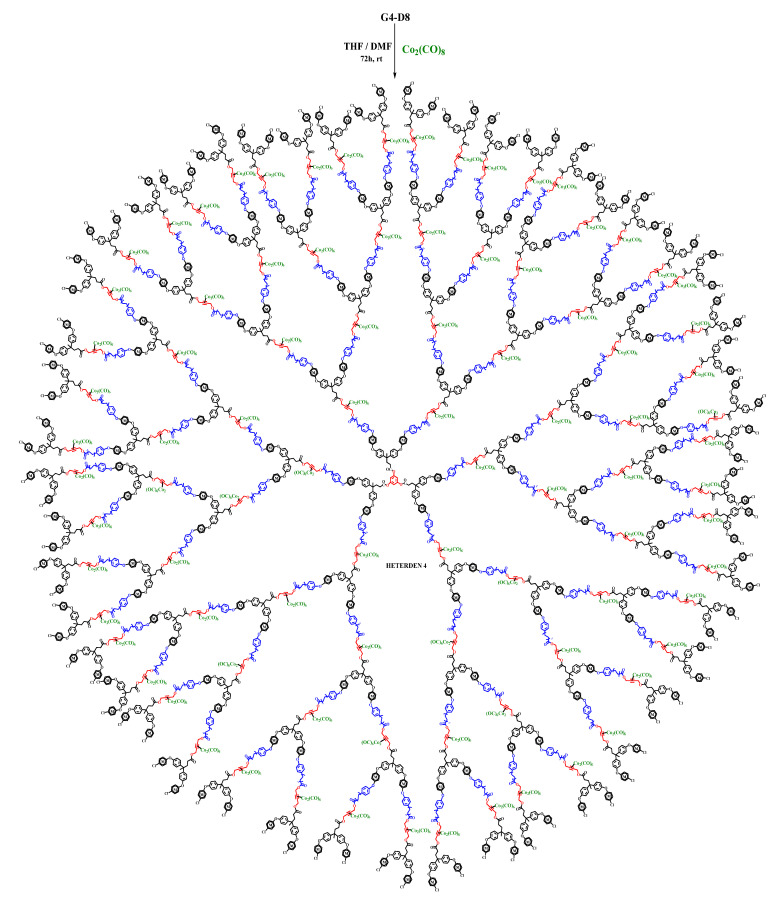
The synthesis of Co/Fe-dendrimer D9 (**HETERODEN 4**).

As described above, reacting carboxylic-end dendrimers with complex **2** resulted in peripheral chloro- end group dendrimers containing alkyne groups (Scheme 1, Scheme 2, Scheme 3 and Scheme 4). The free chloro-terminal group advantages allow further functionalization, whereas alkyne moiety can permit post-modification with a second metallic moiety. Four generations of a heterometallic dendrimer (**HETERODEN1**, **HETERODEN2**, **HETERODEN3**, and **HETERODEN4**) were synthesized via a three-step synthetic procedure. First, **HETERODEN1** was synthesized via nucleophilic substitution reaction of dendrimer **G1–D1** with 5-(4-hydroxyphenyl) pentanoic acid in the presence of K_2_CO_3_ as a catalyst to obtain **G1–D2**; which upon Steglich esterification reaction with complex **2** yielded the **G2–D3** (Scheme 1). In the final step, dicobalt hexacarbonyl was incorporated into **G1–D3** via exploiting the alkyne bonds’ reactivity with cobalt octacarbonyl (Scheme 5). The reaction was left to stir for 72 h to incorporate Co_2_(CO)_6_ completely. Higher generations of heterometallic dendrimers (**HETERODEN2**, **HETERODEN3** and **HETERODEN4**) were synthesized following similar procedures (Scheme 6, Scheme 7 and Scheme 8).

The success of the synthesis of heterometallic dendrimers was monitored using ^1^H and ^13^C NMR spectroscopies and IR spectroscopy and elemental analyses. For example, the ^1^H NMR spectrum of homometallic dendrimer **G1–D2** showed one peak at 6.24 ppm due to the equivalent environment of the iron-complexed arene ligand, which indicates a successful nucleophilic aromatic substitution reaction [40,41]. Furthermore, the resonance of carboxylic acid proton disappeared from the ^1^H NMR spectrum of **G2–D3**, indicating successful esterification between the complex **2** and **G1–D2**. After coordinating Co_2_(CO)_6_ into **G2–D3**, the ^1^HNMR of **HETERODEN1** reveals that the methylene protons resonance shifted downfield from 4.76 ppm to 5.62 ppm due to electron deshielding caused by the cobalt [42,43]. This finding is consistent with data obtained previously [41].

**Figure 1:**
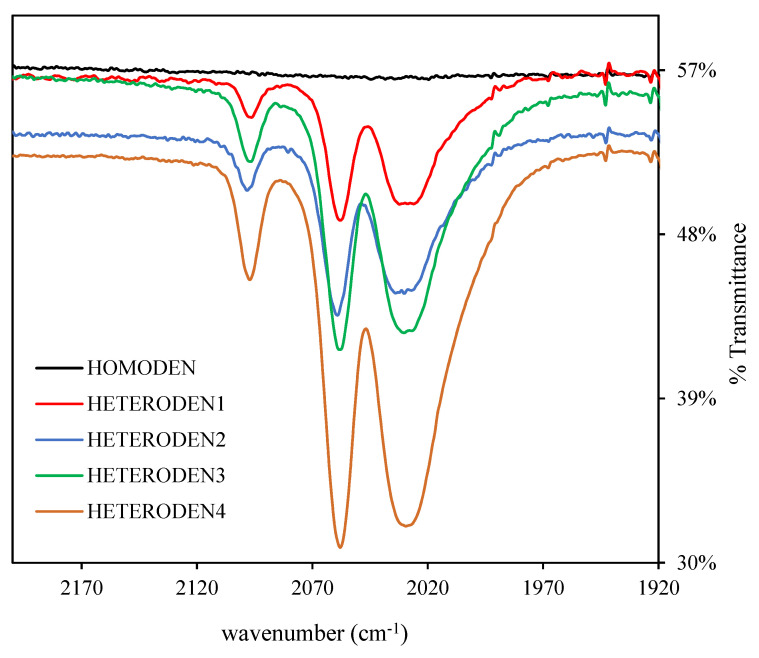
ATR‒FTIR spectra of heterometallic dendrimers indicate CO stretch bands at 2097, 2058, and 2030 cm^−1^ due to coordinated Co_2_(CO)_6_.

**Figure 2:**
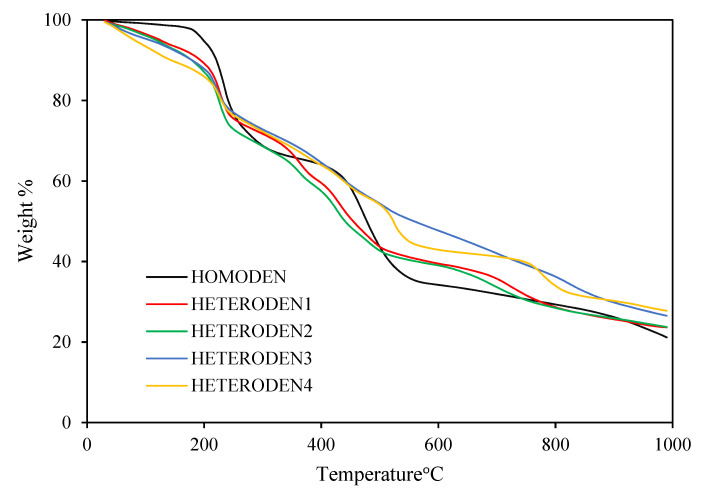
TGA thermogram of the heterometallic dendrimers compared to homometallic dendrimer recorded under nitrogen at a heating rate of 10 °C/min.

Again, as indicated in previous reports [30,41,42,43], carbonyl carbons of the Co_2_(CO)_6_ moieties were detected at 193.71ppm in the ^13^C NMR spectrum of **HETERODEN1**. Additionally, it is interesting to mention that in the ^13^C NMR spectrum of **HETERODEN1**, the resonance of all cobalt-coordinated carbons was broadened [41]. Thus, these carbons were not visible in the ^13^C NMR spectrum due to this broadening. These observations suggested that Co_2_(CO)_6_ was successfully incorporated into the dendrimer branches. It is important to note that high generations of the heterometallic dendrimers (**HETERODEN2**, **HETERODEN3**, and **HETERODEN4**) were partially soluble in common laboratory solvents. Therefore, their characterization using ^1^H and ^13^C NMR was precluded, but we successfully obtained ATR-FTIR spectra and CH analysis data to ensure their successful synthesis. For example, in the ATR-FTIR spectrum of heterometallic dendrimers, three characteristic bands for the CO stretching at 2097, 2057, and 2030 cm^−1^, were observed as previously mentioned [43]. All carbons in these three peaks indicated Co_2_(CO)_6_ presence in the heterometallic dendrimers (Figure 1). The thermal analysis of the **HOMODENs** and **HETERODENs** was conducted using thermogravimetric analysis (TGA) under nitrogen at a heating rate of 10 °C/min. From the TGA thermogram, it is evident that the **HETERODEN1**–**HETERODEN4** began to degrade at 80 °C (Figure 2), due to the decomposition of the alkyne-hexacarbonylcobalt moieties Co_2_(CO)_6_, which is lower than the starting temperature of 200 °C for the same dendrimers without Co_2_(CO)_6_. These findings confirmed that Co_2_(CO)_6_ was successfully coordinated to the dendrimers.

### 2.3. Characterization of Magnetic Particles Containing TMPs Phases

Pyrolysis of the homometallic and heterometallic dendrimers in a tube furnace at 900 °C in an inert atmosphere resulted in ferromagnetic materials containing multimetallic phosphide phases in a small yield (30%). The low yield can be explained by the fact that a large portion of the dendrimers consists of flexible aliphatic fragments, which evaporate at high temperatures as gases [41]. Powder X-ray diffraction (XRD), SEM and EDX, BF-STEM, TEM, and HRTEM were used to gain insight into the morphology and bulk composition of the produced magnetic materials.

#### 2.3.1. X-ray Powder Diffraction (XRD)

**Figure 3:**
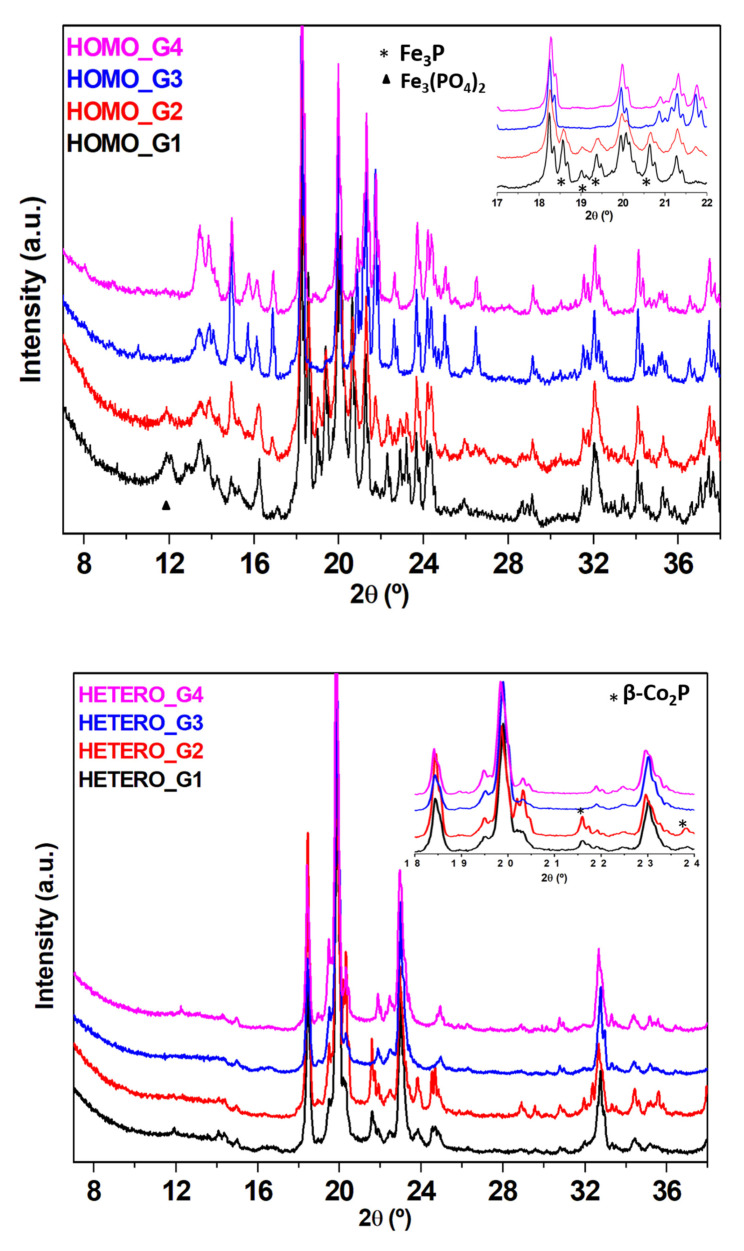
XRD patterns of homometallic (**top**) and heterometallic (**bottom**) magnetic materials. Insets at the right-top corner show a magnification of the angular range where most intensity Bragg peaks appear (The main different peaks are labeled).

X-ray powder diffraction (XRD) patterns of magnetic particles exhibit the XRD pattern of the homometallic (top) and heterometallic (bottom) (Figure 3). The high number of Bragg peaks observed, especially on the homometallic materials, denotes that the samples are multiphasic complexes. In order to clarify the type and amount of the distinct crystalline phases contained in the samples, the whole pattern profile fit (WPPF) of every XRD pattern has been performed using the Rietveld method (RM) (see Figures S1 and S2). In this sense, Tables S1 and S2 show the refined lattice parameters of the different structures used in the WPPF for homometallic and heterometallic particles, respectively. Further, weight percentages and for these crystalline phases identified on each magnetic sample are listed in Table 1 and Table 2. In contrast to the amorphous dendrimer precursor, the magnetic materials were polycrystalline, consisting of various nanocrystals embedded in an amorphous matrix. The X-ray powder diffraction technique [44] shows that the heterometallic samples were more crystalline than homometallic ones, with degrees of crystallinity, ranging from 75 to 86%. The crystals were large as indicated by several sharp peaks around 2*θ* = 16–36° in the diffractograms, and their sizes were greater than 50 Å as determined by the Scherrer equation [45] (Tables S3 and S4).

Concerning homometallic samples, the structural characterization derived from XRD patterns analysis shows that the magnetic particles are formed mainly by iron phosphides and phosphates. Only a few weight percentages (below 5%) of metallic iron was determined for the first- and second-generation complexes. On the other hand, the heterometallic samples present a higher number of metallic structures, which include close-packed structures as face-centered cubic (FCC), hexagonal close-packed (HCP) and body-centered cubic (BCC). Besides that, about 35–50% wt of cobalt phosphide (2/1) is also established in both forms. It is worth noting that every structural determination is focused only on crystalline phases, so amorphous material such as the carbon matrix or even other phases not identified were not quantified.

#### 2.3.2. Electron Microscopy Studies

**Figure 4:**
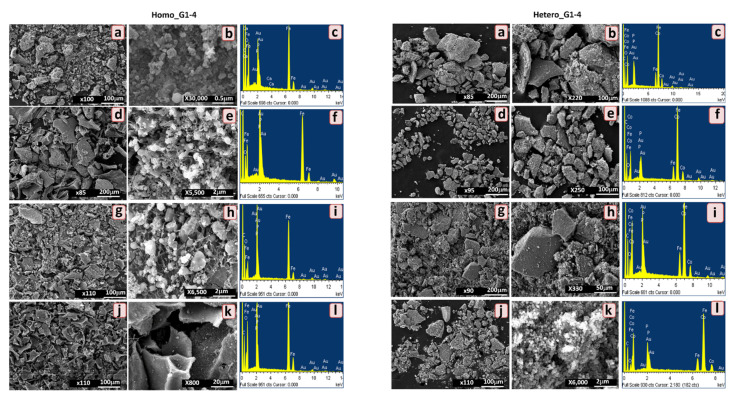
SEM images and EDS spectra for homometallic (**left**) and heterometallic (**right**); **HOMO-G1/HETERO-G1** (**a**–**c**), **HOMO-G2/HETERO-G2** (**d**–**f**), **HOMO-G3/HETERO-G3** (**g**–**i**) and **HOMO-G4/HETERO-G4** (**j**–**l**) samples, respectively.

**Figure 5:**
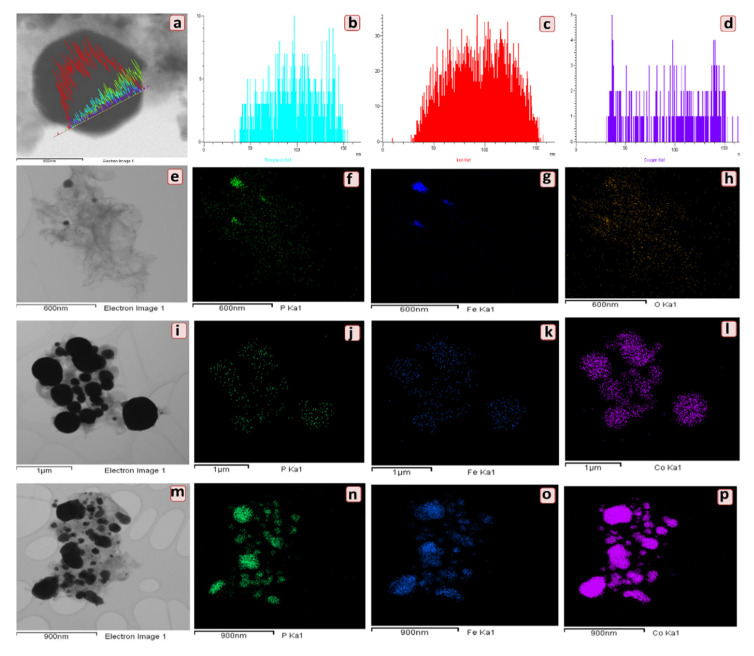
BF-STEM elemental mapping for **HOMO-G1** (**a**–**d**), **HOMO-G3** (**e**–**h**), **HETERO-G2** (**i**–**l**) and **HETERO-G3** (**m**–**p**) samples; P (**b**,**f**,**j**,**n**), Fe (**c**,**g**,**k**,**o**), O (**d**,**h**) and Co (**l**,**p**) elemental maps.

**Figure 6:**
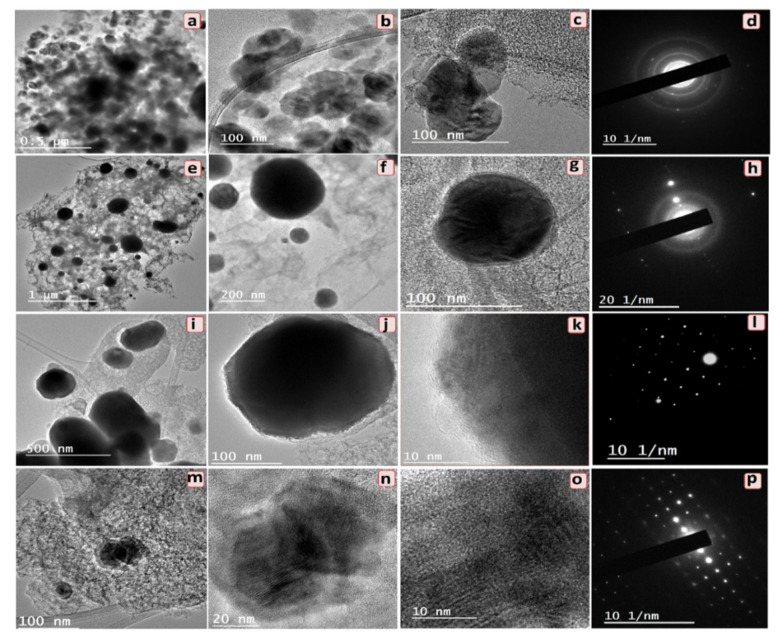
TEM and HRTEM micrographs for **HOMO-G1** (**a**–**c**), **HOMO-G3** (**e**–**g**), **HETERO-G2** (**i**–**k**) and **HETERO-G3** (**m**–**o**) samples and SAED of polycrystalline **HOMO-G1** and **HOMO-G3** particles shown in (**d**,**h**) and single-crystalline ones in (**l**,**p**).

The chemical composition, morphology, and particle size distribution of homometallic and heterometallic samples have been investigated by SEM and EDS analysis (Figure 4), which verified the plate-like morphology of the particles with an extensive size distribution. Also, it confirmed the presence of iron, phosphorus, and oxygen for the homometallic samples and the additional doped cobalt for heterometallic ones. The homometallic **G1** and **G3**, heterometallic **G2** and **G3** samples were selected for BF-STEM, TEM, and HRTEM studies. The elemental mapping (Figure 5) emphasizes the heterogeneous dispersion of the EDS detected elements of two systems. Overall, these results are highly consistent with the PXRD analysis. TEM and HRTEM micrographs (Figure 6) of samples indicate the crystalline nature of the nearly aggregated particles. Selected area electron diffraction (SAED) patterns (Figure 6d,h,l,p) exhibited a polycrystalline nature of homometallic samples and reflected single-crystalline diffraction for heterometallic ones.

#### 2.3.3. Magnetic Properties

**Figure 7:**
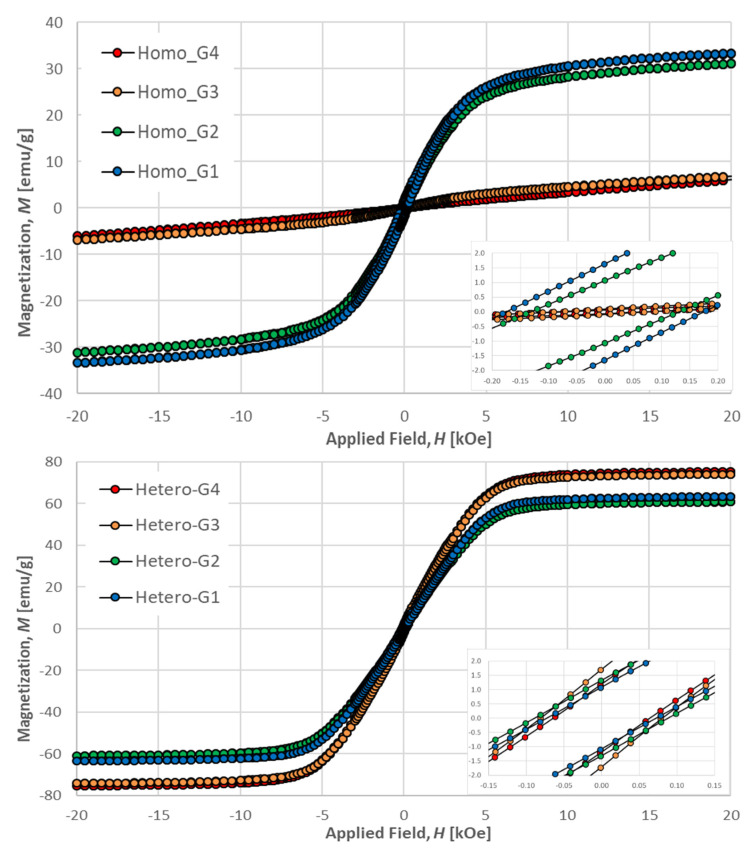
Room temperature hysteresis loops for the homometallic (**top**) and heterometallic (**bottom**) samples. Insets at the right‒bottom corner show a magnification of the central part of the cycles.

An initial examination of the magnetic properties of particles using a magnetic stir bar indicated some kind of inducted magnetism because the samples were attracted to the magnetic bar. To further understand their magnetic properties, an EV9 Microsense Vibrating Sample Magnetometer (VSM) was used to get the magnetization curve (Figure 7), which depicts the magnetization vs. applied magnetic field *M*(*H*) measured at room temperature (RT) for homometallic (top) and heterometallic (bottom) materials. Except for **G3** and **G4** on homometallic samples, the rest of the samples exhibit a typical hysteresis loop characteristic of ferromagnetic substances, which separate increasing and decreasing field branches. In this way, the absence of a hysteresis loop in **G3** and **G4** of homometallic samples could be due mainly to the absence of metallic iron as confirmed by XRD results (Table 1). In addition, magnetic properties of the iron phosphides observed in homometallic particles are significantly different according to their stoichiometry and structure. Thus, the FeP orthorhombic phase exhibits antiferromagnetic properties below 115 K, whereas the tetragonal (hexagonal) crystal structures of Fe_3_P (Fe_2_P) phase shows ferromagnetic temperature above (below) RT [46,47]. So, it is reasonable to assume that the absence of both Fe and Fe_3_P phases could be contributed to the lack of the hysteresis loop on the former samples.

The remanent magnetization (*M_r_*) and coercive field (*H_c_*) of these ferromagnetic complexes are directly determined in *M*(*H*) curves from the magnetization and field axis crossing points, respectively, whereas the saturation magnetization (*M_s_*) is estimated through the maximum magnetization value reached (Table 3 and Table 4).

Concerning this ferromagnetic parameter, saturation magnetization values seem to be correlated with the metallic amount present in each sample. Since the heterometallic particles have more metallic phases than homometallic particles, this parameter reaches higher values than the ferromagnetic homometallic ones and ultimately levels off saturation magnetization (*M_s_*) of 60–75 emu/g are reached on this group. Besides that, the homometallic particles showed dendritic effects on ferromagnetic parameters, particularly *H_c_* and *M_s_*, which decreased with increased generations [41]. Moreover, the magnetic properties of nanoparticles are governed by various parameters, including concentration and interparticle distance [48,49,50,51]. Thus, it has been previously shown that as the concentration of magnetic species increases to a critical point, the distance between particles decreases, which leads to an increase in dipolar interaction and eventually an increase in *H_c_* [51,52,53]. Therefore, it is logical to presume that changes in iron concentration in the homometallic samples regulated *H_c_* and *M_s_*, leading to the observed trend. In contrast to the homometallic particles, no structure-property relationship was observed within the heterometallic ones.

**Figure 8:**
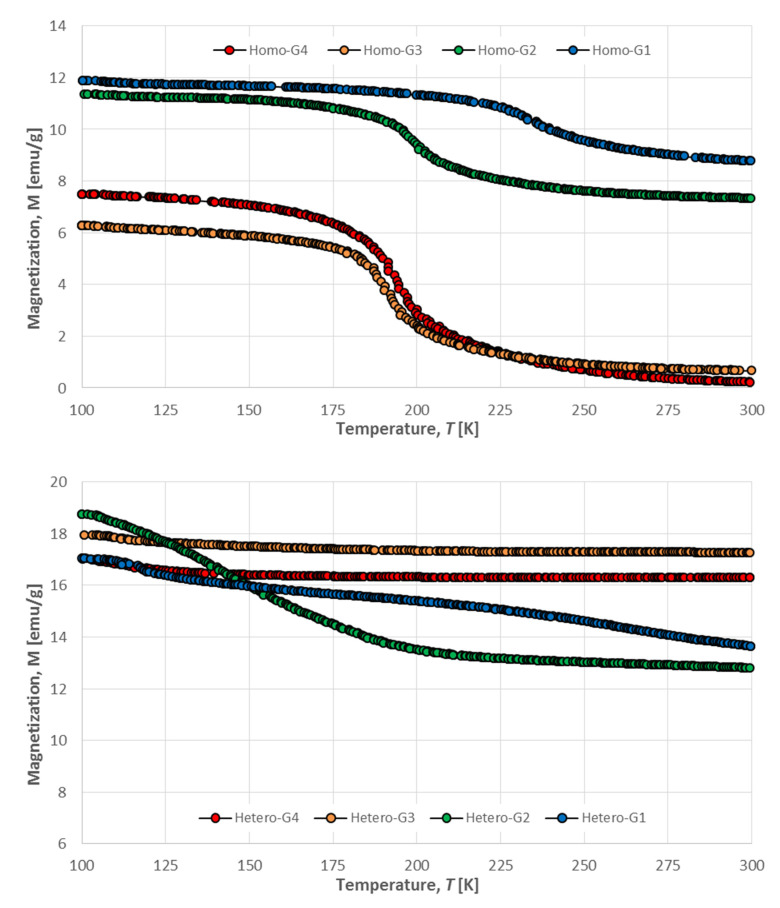
Temperature-dependent magnetization curves for the homometallic (**top**) and heterometallic (**bottom**) samples measured.

On the other hand, Figure 8 shows the temperature dependence of magnetization, *M*(*T*) curves, under an applied magnetic field of 1 kOe. The *M*(*T*) curves for homometallic samples exhibit an offset in their magnetization at temperatures around to 200 K, a step which seems to be correlated with Fe_2_P percentages (Table 1). Moreover, the iron phosphide (2/1) Curie temperature of 209 K could be slightly altered by means of stoichiometry, interparticle interaction, morphology or structure [48]. In this sense, orthorhombic cobalt phosphides (2/1) were not ferromagnetic, in contrast with the hexagonal iron phosphide (2/1). Nevertheless, the latter phase is isostructural to the beta-form of the cobalt phosphide (2/1) so a possible doping of iron in this form could be on the slope change observed on *M*(*T*) curves for the heterometallic samples with β-Co_2_P content (i.e.,: **Hetero-G1** and **G2**). Finally, it is clear in the figure that the larger cobalt-iron nanoclusters in heterometallic samples have helped to enhance their magnetization susceptibility significantly and also stabilize the magnetization temperature dependence compared with homometallic samples.

## 3. Materials and Methods

### 3.1. Materials and Instrumentation

All used chemicals and reagents were purchased from Sigma-Aldrich and utilized as received. The solvents were dried and stored over 3 Å molecular sieves before being used. The designs of the organoiron complex **1** and dendrimer **G1–D1** were achieved by following previously reported procedures [54,55,56]. Morphological characterizations, PXRD diffraction, and magnetic properties studies were performed at the University of Oviedo-CINN, Spain.

### 3.2. Instrumentation

All NMR spectra of synthesized dendrimers were recorded using a Bruker Avance NMR Spectrometer (^1^H, 300 MHz and ^13^C, 75 MHz). The spectra were obtained from a DMSO-d6 solution of the complexes with the chemical signals referenced to the solvent residual signal in ppm. Attenuated total reflection Fourier-transform IR (ATR-FTIR) spectroscopic measurements were achieved on a Bruker Alpha FTIR spectrometer Alpha-P using KBr pellet technique in the wavenumber range between 4000 to 400 cm^−1^. Elemental carbon and hydrogen analyses for prepared dendrimers were performed on CE-440 Elemental Analyser, Exeter Analytical. Thermogravimetric analysis (TGA) was conducted in platinum pans under nitrogen at a heating rate of 10 °C on a TA Instruments TGA Q500.

#### 3.2.1. Morphological Characterization

SEM micrographs and X-ray microanalysis (SEM/EDX) were recorded using a JEOL-6610 LV scanning electron microscope operating at 30 kV coupled with an Oxford X-Max microanalysis system (EDX). TEM and BF-STEM images, SAED patterns, and BF-STEM/EDX were recorded on a JEOL JEM-2100F field emission transmission electron microscope, at 200 kV, equipped with a STEM unit and an Oxford X-Max microanalysis system (EDX).

#### 3.2.2. X-ray Diffraction

The X-ray diffraction (XRD) patterns for magnetic materials were measured on a Seifert XRD 3000 TT diffractometer operating in Bragg-Brentano geometry. Measurements were done using MoK_α_ radiation (0.7107 Å), where the primary incident, secondary anti-scatter slits of 2 mm and a primary mask were used to illuminate the powder samples compacted in a 30 mm square plastic holder. Vertical Soller slits were also used after the incident and anti-scatter slits to delimit the axial divergence. A secondary high-orientated pyrolytic graphite (HOPG) monochromator and 0.2 mm receiving slit were placed before the scintillation detector. XRD patterns were collected between 7° and 38° 2θ range in step mode, recording diffracted intensity every 0.02° with 20 s counting time for each point.

#### 3.2.3. Magnetometry

Room temperature (RT) magnetization curves, *M*(*H*), were measured in a EV9 Microsense Vibrating Sample Magnetometer (VSM) within an applied magnetic field range of ±20 kOe. Besides that, VSM is equipped with EV1-LNA temperature control option, which allows us to establish the temperature dependence of magnetization, *M*(*T*) curve, under 1 kOe applied magnetic field in a 10 K/min heating rate from 100 to 300 K. In order to perform both measurements, about 100 mg of each complex were encapsulated into a 70 mm^3^ acrylic cup which is firmly stuck to an 8 mm Pyrex transverse rod by means double side Scotch^®^ and PTFE tapes.

### 3.3. Synthesis and Characterization

#### 3.3.1. General Procedure

Five generations of dendrimers were synthesized in a good yield (73–87%). Incorporating the triple bond throughout dendrimer branches was achieved by reacting the carboxylic group in complex **1** with the hydroxyl group in the compound **a** in the presence of DMAP and DCC to give complex **2**, which is based on nucleophilic aromatic substitution reaction (S_N_Ar) with **G1–D1** in the presence of the weak base, K_2_CO_3_ produced **G2–D3**. The synthesis of the five generations of homometallic dendrimers involved a well-established nucleophilic aromatic substitution reaction (S_N_Ar) and an esterification reaction. For instance, the synthesis of homometallic dendrimers **G1–D2**, **G2–D4**, **G3–D6**, and **G4–D8**, involved nucleophilic substitution reaction of homometallic dendrimers **G1–D1**, **G2–D3**, **G3–D5**, **G4–D7** with 5-(4-hydroxyphenyl)-pentanoic acid utilizing 1:6, 1:12, 1:24, and 1:48 molar ratios. The reactant mixtures were stirred in DMF with the presence of K_2_CO_3_ at 55 °C for 24 h after flushing with nitrogen for 30 min. Consequently, the mixtures were then poured into a 10% HCl solution, and NH_4_PF_6_ was added to get the final products.

The Steglich esterification procedure was used to synthesize dendrimers **G2–D3**, **G3–D5**, **G4–D7**, and **G5–D9** via reacting dendrimers **G1–D2**, **G2–D4**, **G3–D6**, **G4–D8** with complex **2** using appropriate molar ratios. First, the reaction mixtures were stirred at 0 °C for 10 min. After that, the mixtures were stirred at room temperature under nitrogen for 15 min before being left to stir for another two days at room temperature. Purification was achieved by maintaining the mixture at −25 °C for two hours, filtered to remove the remaining DHU, and the removal of the solvent gave rise to the products. Detailed synthetic methodologies, yields %, and characterization using ^1^H and ^13^C NMR, ATR-FTIR, and elemental analyses data are reported here.

#### 3.3.2. Synthesis of Complex **2**, from 2-Butyne-1,4-Diol (a) with Complex **1**

Steglich esterification was used to react the carboxylic group in complex **1** with the hydroxyl group in the 2-butyne-1.4-diol to synthesize complex **2** [57]. In 25 mL round-bottom flask was added complex **1** (0.50 g, 0.46 mmol), 2-butyne-1.4-diol (0.04 g, 0.46 mmol), DMAP (0.12 g, 0.95 mmol), 10 mL of DMF as solvent, and then DCC (0.09 g, 0.46 mmol). (Molecular weight 1107.28 g/mol). Yield: (0.41 g, 93%). ^1^HNMR data δ_H_ (300 MHz; DMSO-d_6_): 7.40 (4H, d, *J* = 7.8 Hz, uncompleted Ar-H), 7.29 (4H, d, *J*= 8.1 Hz, uncompleted Ar-H), 6.83 (4H, d, *J* = 6.3 Hz, complexed Ar-H), 6.42 (4H, s, complexed Ar-H), 5.29 (10H, s, Cp-H), 5.31 (H, s, OH), 4.72 (2H, s, CH_2_), 4.11 (2H, s, CH_2_), 2.43 (2H, s, CH_2_), 2.23 (2H, s, CH_2_), 1.68 (3H, s, CH_3_). ^13^C NMR *δ*_c_ (75 MHz; DMSO-d_6_): 173.02 (CO), 154.35, 152.43, 147.28, 146.97, 132.86, and 104 (quat-C), 130.21 and 121.04 (uncomplexed Ar-C), 87.68 and 77.29 (complexed Ar-C), 80.22 (Cp-C), 52.92 and 49.76 (C≡C), 50.28, 33.84, and 31.28 (CH_2_), 27.89 (CH_3_). ATR-FTIR; *ν_max_*/cm^−1^: 3339 (OH), 2939 (Ar-C), 1726 (CO), 1229 (C-O-C). Elemental analysis for C_43_H_38_O_5_Cl_2_Fe_2_P_2_F_6_: calculated %C 46.64, %H 3.46, and found %C 47.01, and %H 3.43.

#### 3.3.3. Carboxylic-Terminated G1-Homometallic Dendrimer (**G1–D2**)

Nucleophilic substitution reaction was used to create **G1–D2**. In 25 mL round-bottom flask was added **G1–D1** (0.25 g, 0.08 mmol), compound **a** (0.09 g, 0.5 mmol), K_2_CO_3_ (0.32 g, 2.30 mmol), and 10 mL of DMF as solvent. (Molecular weight 4136.27 g/mol). Yield: (0.26 g, 82%). ^1^H NMR (300 MHz; DMSO-d_6_): 12.06 (6H, s, COOH), 7.38 (27H, d, *J* = 8.4, Hz, uncomplexed Ar-H), 7.24 (24H, d, *J*= 8.4Hz, uncomplexed Ar-H), 6.23 (24H, s, complexed Ar-H), 5.21 (30H, s, Cp-H), 2.64 (18H, s, CH_2_), 2.39(12H, s, CH_2_), 2.26 (6H, s, CH_2_), 2.10(24H, s, CH_2_),1.61 (9H, m, CH_3_). ^13^C NMR *δ*_c_ (75 MHz; DMSO-d_6_,): 175.48 (CO), 159.80, 152.12, 146.89, 141.36, 139.51, and 131.29 (quat-C), 131.30, 130.57, 130.05, 128.79, 122.27, 121.30, 119.77, and 115.85 (uncomplexed Ar-C), 78.70 (Cp-C), 76.97 and 75.45 (complexed Ar-C), 45.92, 34.34, 33.57, and 31.64 (CH_2_), 27.92 (CH_3_). ATR-FTIR; *ν_max_*/cm^−1^: 3312 (COOH), 2935 (Ar-CH), 2876 (Cp-CH), 1745 (CO), 1218 (C-O-C). Elemental analysis for C_189_H_180_O_30_Fe_6_P_6_F_36_: calculated %C 54.83, %H 4.35, and found %C 55.10, and %H 4.63.

#### 3.3.4. Chloro-Terminated G2-Homometallic Dendrimer (**G2–D3**)

**G1–D2** was used with complex **2** to synthesize dendrimer **G2–D3**, following the Steglich esterification procedure analogous to the synthesis of complex **2**. In a 25 mL round-bottom flask (0.40 g, 0.36 mmol) of complex **2**, (0.25 g, 0.06 mmol) of **G1–D2**, (0.17 g, 1.39 mmol) of DMAP were mixed in 10 mL of DMF as solvent, while DCC (0.14 g, 0.72 mmol) was added over a 15 min. (Molecular weight 10,671.85 g/mol). Yield: (0.55 g, 85%). ^1^H NMR (300 MHz; DMSO-d_6_): 7.36 (51H, s, uncomplexed Ar-H), 7.28 (48H, d, *J* = 8.4 Hz, uncomplexed Ar-H), 6.81 (24H, d, *J* = 6 Hz, complexed Ar-H), 6.41 (24H, s, complexed Ar-H), 6.23 (24H, s, complexed Ar-H), 5.28 (60H, s, Cp-H), 5.21 (30H, s, Cp-H), 4.76 (24H, s, CH_2_), 2.43 (42H, s, CH_2_), 2.23(18H, s, CH_2_), 2.16 (24 H, s, CH_2_), 1.68 (27H, s, CH_3_). ^13^C NMR *δ*_c_ (75 MHz; DMSO-d_6_,): 172.71, and 170.25 (CO), 157.50, 154.27, 152.12, 147.51, 132.79, and 104.31 (quat-C), 134.18, 133.03, 131.26, 130.74, 130.14, 129.59, 122.26, 121.27, 121.01, 120.78, and 119.24 (uncomplexed Ar-C), 80.21, 78.69 (Cp-C), 87.67, 77.27, 76.01, and 75.45 (complexed Ar-C), 52.72 and 49.82 (C≡C), 46.01, 38.17, 36.73, 34.32, 33.69, and 31.57 (CH_2_), 27.83 (CH_3_). ATR-FTIR; *ν_max_*/cm^−1^: 2938 (Ar-CH), 2825 (Cp-CH), 1725 (CO), 1221 (C-O-C). Elemental analysis for C_447_H_396_O_54_Cl_12_Fe_18_P_18_F_108_: calculated %C 50.26, %H 3.71, and found %C 50.87, and %H 4.16.

#### 3.3.5. Carboxylic Acid-Terminated G2-Homometallic Dendrimer (**G2–D4**)

Dendrimer **G2–D4** was synthesized from **G2–D3** and compound **a** following nucleophilic substitution reaction. In a 25 mL round-bottom flask (0.23 g, 0.02 mmol) of **G2–D3**, (0.05g, 0.25 mmol) of compound **a**, (0.18 g, 1.28 mmol) K_2_CO_3_ were mixed in 10 mL of DMF as solvent. (Molecular weight 12,565.05 g/mol). Yield: (0.21 g, 73%). ^1^H NMR data δ_H_ (300 MHz; DMSO-d_6_): 12.04 (12H, s, COOH), 7.35 (75H, s, uncomplexed Ar-H), 7.24 (72H, s, uncomplexed Ar-H), 6.24 (72H, s, complexed Ar-H), 5.21 (90H, s, Cp-H), 4.76 (24H, s, CH_2_), 2.36 (90H, s, CH_2_), 2.25 (18H, s, CH_2_), 2.10 (72H, s, CH_2_), 1.65 (27, s, CH_3_). ^13^C NMR *δ*_c_ (75 MHz; DMSO-d_6_,): 175.48, 173.33, and 170.25 (CO), 157.65, 154.27, 152.02, 146.54, 143.44, 141.10, and 132.02 (quat-C), 131.46, 131.28, 130.57, 130.02, 129.42, 122.30, 121.28, 120.75, 119.05, 118,37, 115.84 and 107.25 (uncomplexed Ar-C), 78.68 (Cp-C), 76.01 and 75.45 (complexed Ar-C), 52.72 and 49.79(C≡C), 50.26, 48.36, 36.38, 33.47, and 31.83 (CH_2_), 28.01 (CH_3_). ATR-FTIR; *ν_max_*/cm^−1^: 3316 (COOH), 2928 (Ar-CH), 2856 (Cp-CH), 1724 (CO), 1219 (C-O-C). Elemental analysis for C_579_H_552_O_90_Fe_18_P_18_F_108_: calculated %C 55.29, %H 4.39, and found %C 55.58, and %H 4.54.

#### 3.3.6. Chloro-Terminated G3-Homometallic Dendrimer (**G3–D5**)

**G2–D4** was used with complex **2** to synthesize **G3–D5**, following the Steglich esterification procedure. In a 25 mL round-bottom flask (0.26 g, 0.24 mmol) of complex **2**, (0.25 g, 0.02 mmol) of **G2–D4**, (0.12 g, 0.95 mmol) of DMAP were mixed in 10 mL of DMF as solvent. Then DCC (0.09 g, 0.47 mmol) was added over 15 min. The product resulted in a yellow solid (Molecular weight 25,602.18 g/mol). Yield: (0.45 g, 87%). ^1^H NMR (300 MHz; DMSO-d_6_): 7.35 (123H, d, *J* = 6.6 Hz, uncomplexed Ar-H), 7.23 (120H, t, *J* = 8.7 Hz, 7.5 Hz, uncomplexed Ar-H), 6.80 (48H, d, *J* = 6.6 Hz, complexed Ar-H), 6.43 (48H, d, *J* = 4.8 Hz, complexed Ar-H), 6.23 (72, s, complexed Ar-H), 5.28 (120H, s, Cp-H), 5.21 (90H, s, Cp-H), 4.77 (72H, s, CH_2_), 2.41 (78H, s, CH_2_), 2.26 (42H, s, CH_2_), 2.18 (72H, s, CH_2_),1.68 (36H, s, CH_3_), 1.63 (63H, s, CH_3_). ^13^C NMR *δ*_c_ (75 MHz; DMSO-d_6_,): 173.02, and 170.25 (CO), 154.27, 152.12, 144.08, 132.39, 111.86, and 104.31 (quat-C), 134.18, 133.03, 131.26, 130.55, 130.1, 121.01, 119.04, and 109.97 (uncomplexed Ar-C), 80.21, 78.68 (Cp-C), 87.66, 77.25, and 75.97 (complexed Ar-C), 51.85, and 49.34 (CC), 34.42, 32,88, 30.73 and 28.01 (CH_2_), 27.80 (CH_3_). ATR-FTIR; *ν_max_*/cm^−1^: 2928 (Ar-CH), 2853 (Cp-CH), 1730 (CO), 1225 (C-O-C). Elemental analysis for C_1095_H_984_O_138_Cl_24_Fe_42_P_42_F_252_: calculated %C 51.32, %H 3.84, and found %C 51.78, and %H 4.10.

#### 3.3.7. Carboxylic Acid-Terminated G3-Homometallic Dendrimer (**G3–D6**)

**G3–D6** was synthesized from **G3–D5** (0.25 g, 0.009 mmol), compound **a** (0.04 g, 0.23 mmol), K_2_CO_3_ (0.16 g, 1.16 mmol), in 10 mL of DMF through nucleophilic substitution reaction. The mixture was left stirring at 55 °C for 24 h after flushing with nitrogen for 30 min. (Molecular weight 29,422.59 g/mol). Yield: (0.24 g, 85%). ^1^H NMR (300 MHz; DMSO-d_6_): 12.04 (24H, s, COOH), 7.38 (171H, d, *J* = 8.4Hz uncomplexed Ar-H), 7.24 (168H, d, *J* = 8.1 Hz uncomplexed Ar-H), 6.23 (168H, s, complexed Ar-H), 5.21 (210H, s, Cp-H), 4.76 (72H, s, CH_2_), 2.61 (210H, s, CH_2_), 2.35 (42H, s, CH_2_), 2.24 (168H, s, CH_2_),1.71 (63H, m, CH_3_). ^13^C NMR *δ*_c_ (75 MHz; DMSO-d_6_,): 175.78, 173.32, and 170.25 (CO), 157.65, 152.43, 146.89, 141.05, and 131.70 (quat-C), 131.28, 130.71, 130.58, 130.04, 128.80, 122.31, 121.28,120.76, and 115.83 (uncomplexed Ar-C), 78.68, and 77.86 (Cp-C), 76.36, 76.03, 75.45, and 73.57 (complexed Ar-C), 52.39, and 48.47 (C≡C), 45.82, 37.24, 36.74, 35.03, 33,11, and 31.03 (CH_2_), 27.94 (CH_3_). ATR-FTIR; *ν_max_*/cm^−1^: 3305 (COOH), 2928 (Ar-CH), 2861 (Cp-CH), 1732 (CO), 1224 (C-O-C). Elemental analysis for C_1359_H_1296_O_210_Fe_42_P_42_F_252_: calculated %C 55.42, %H 4.40, and found %C 56.12, and %H 4.64.

#### 3.3.8. Chloro-Terminated G4-Homometallic Dendrimer (**G4–D7**)

In a process similar to synthesis of **G2–D3**, **G4–D7** was synthesized from **G3–D6** (0.25 g, 0.008 mmol), complex **2** (0.22 g, 0.20 mmol), DMAP (0.089 g, 0.81mmol), and DCC (0.08 g, 0.40 mmol), in 10 mL of DMF. The producing yellow solid (Molecular weight 55564.91 g/mol) was collected by suction filtration and left to dry under vacuum for two days. Yield: (0.45 g, 87%). ^1^H NMR data (300 MHz; DMSO-d_6_): 7.35 (267H, s, uncomplexed Ar-H), 7.29 (264H, t, *J* = 8.4 Hz, 7.8Hz uncomplexed Ar-H), 6.82 (96H, d, *J* = 6.3 Hz, complexed Ar-H), 6.44 (96H, m, complexed Ar-H), 6.23 (168H, s, complexed Ar-H), 5.28 (240H, s, Cp-H), 5.21 (210H, s, Cp-H), 4.77 (168H, s, CH_2_), 2.59 (258H, s, CH_2_), 2.41 (90H, s, CH_2_), 2.18 (168H, s, CH_2_),1.68 (135H, m, CH_3_). ^13^C NMR *δ*_c_ (75 MHz; DMSO-d_6_,): 175.32, 172.71, and 170.47 (CO), 164.80, 157.35, 154.58, 152.19, 147.34, 141.16, 132.89, 131.64, and 104.31 (quat-C), 131.28, 130.56, 130.13, 122.30, 121.29, 121.0, and 120.77 (uncomplexed Ar-C), 80.22 and 79.51 (Cp-C), 87.67, 77.93, 77.26, 75.99, 75.44, and 73.68 (complexed Ar-C), 52.77, and 48.36 (C≡C), 45.18, 38.08, 37.11, 35.60, 33.34, 32.77 and 31.24 (CH_2_), 27.80 (CH_3_). ATR-FTIR; *ν_max_*/cm^−1^: 2936 (Ar-CH), 2856 (Cp-CH), 1732 (CO), 1223 (C-O-C). Elemental analysis for C_2391_H_2160_O_306_Cl_48_Fe_90_P_90_F_540_: calculated %C 51.63, %H 3.88, and found %C 52.10, %H 4.21.

#### 3.3.9. Carboxylic Acid-Terminated G4-Homometallic Dendrimer (**G4–D8**)

**G4–D8** was synthesized by following same synthesis process of **G1–D2**, from **G4–D7** (0.25 g, 0.02 mmol), **a** (0.04 g, 0.22 mmol), K_2_CO_3_ (0.15 g, 1.07 mmol), in 10 mL of DMF. The product was yellow solid collected by suction filtration and dried under vacuum at room temperature (Molecular weight 63,137.68 g/mol). Yield: (0.21 g, 73%). ^1^H NMR data δ_H_ (300 MHz; DMSO-d_6_): 12.04 (48H, s, COOH), 7.37 (363H, d, *J* = 8.1 Hz uncomplexed Ar-H), 7.24 (360H, d, *J* = 8.1 Hz uncomplexed Ar-H), 6.23 (360H, s, complexed Ar-H), 5.21 (450H, s, Cp-H), 4.77 (168H, d, J = 8.7 Hz, CH_2_), 2.65 (450H, s, CH_2_), 2.39 (90H, s, CH_2_), 2.24 (360H, s, CH_2_), 1.69 (135H, m, CH_3_). ^13^C NMR *δ*_c_ (75 MHz; DMSO-d_6_,): 175.47, 173.78, and 170.80 (CO), 157.36, 154.80, 154.06, 152.12, 148.89, 141.21, and 131.52 (quat-C), 131.27, 130.56, 130.02, 129.41, 122.29, 121.27, 120.74, 119.05, 118.36, and 115.85 (uncomplexed Ar-C), 78.68, and 77.66 (Cp-C), 76.39, 76.02, and 75.46 (complexed Ar-C), 52.43, and 49.38 (C≡C), 45.89, 38.20, 34.91, 33.31, 32.51, 31.21, and 30.35 (CH_2_), 27.94 (CH_3_). ATR-FTIR; *ν_max_*/cm^−1^: 3310 (COOH), 2922 (Ar-CH), 2867 (Cp-CH), 1732 (CO), 1227 (C-O-C). Elemental analysis for C_2919_H_2784_O_450_Fe_90_P_90_F_540_: calculated %C 55.47, %H 4.41, and found %C 55.89, and H 4.76.

#### 3.3.10. Chloro-Terminated G5-Homometallic Dendrimer (**G5–D9**)

In a process similar to the synthesis of **G2–D3**, **G5–D9** was synthesized from **G4–D8** (0.25 g, 0.004 mmol), complex **2** (0.21 g, 0.2 mmol), DMAP (0.09 g, 0.75 mmol), and DCC (0.08 g, 0.37 mmol), in 10 mL of DMF. The yellow solid was collected by using suction filtration at room temperature (Molecular weight 115,422.31 g/mol). Yield: (0.32 g, 75%). ^1^H NMR (300 MHz; DMSO-d_6_): 7.34 (555H, s, uncomplexed Ar-H), 7.28 (192H, s, uncomplexed Ar-H), 7.23 (360H, s, uncomplexed Ar-H), 6.81 (192H, d, *J* = 6.3 Hz, complexed Ar-H), 6.41((192H, s, complexed Ar-H), 6.23 (360H, s, complexed Ar-H), 5.28 (480H, s, Cp-H), 5.21 (450H, s, Cp-H), 4.75 (360H, d, J = 8.7 Hz, CH_2_), 2.41 (546H, s, CH_2_), 2.37 (186H, s, CH_2_), 2.24 (360H, s, CH_2_), 1.68 (279H, m, CH_3_). ^13^C NMR (75 MHz; DMSO-d_6_,): 173.67, 172.24, and 170.25 (CO), 164.26, 157.55, 154.50, 152.43, 152.06 147.28, 147.05, 141.25, 132.77, 115.55, 131.48 and 104.48 (quat-C), 131.27, 130.56, 130.13, 121.28, 121.01, 120.76, and 108.86 (uncomplexed Ar-C), 80.21 and 78.68 (Cp-C), 87.67, 77.27, 76.01, and 75.45 (complexed Ar-C), 53.96, and 51.76 (C≡C), 68.38, 57.59, 53.47, 52.73, 45.93, 36.26, 34.18, 33.55, 32,54, 31.22, and 30.34 (CH_2_), 27.82 (CH_3_). ATR-FTIR; *ν_max_*/cm^−1^: 2923 (Ar-CH), 2854 (Cp-CH), 1728 (CO), 1231 (C-O-C). Elemental analysis for C_4983_H_4512_O_642_Cl_96_Fe_186_P_186_F_1116_: calculated %C 51.81, and %H 3.91, and found %C 52.15, %H 4.34.

#### 3.3.11. Heterometallic Dendrimer Co/Fe-dendrimerd3 (**HETERODEN 1**)

The heterometallic dendrimer was synthesized using a previously reported method [42,58]. Under the N_2_ atmosphere, homometallic dendrimer **G2–D3** (0.25 g, 0.023 mmol) was dissolved in 1 mL of dried DMF and 10 mL of dried THF. Co_2_(CO)_8_ was added upon complete dissolution, and then the reaction mixture was stirred at room temperature inside the glovebox for 72 h. Subsequently, the THF was evaporated using rotary evaporation, and the red residue was dissolved into 100 mL acetone. After 10 min, the brown precipitate formed was filtered and reprecipitated into 10-fold excess of diethyl ether. This technique was used three times to obtain the final product in 72% yield as a brown solid (Molecular weight 12,387.04 g/mol). ^1^H NMR (300 MHz; DMSO-d_6_): 7.47 (51H, s, uncomplexed Ar-H), 7.34 (48H, s, uncomplexed Ar-H), 6.83 (24H, s, complexed Ar-H), 6.52 (24H, s, complexed Ar-H), 6.35 (24H, s, complexed Ar-H), 5.62 (24H, s, CH_2_), 5.40 (60H, s, Cp-H), 5.32 (30H, s, Cp-H), 2.43 (42H, s, CH_2_), 2.26(18H, s, CH_2_), 2.08 (24 H, s, CH_2_), 1.63 (27H, s, CH_3_). ^13^C NMR *δ*_c_ (75 MHz; DMSO-d_6_,): 193.71, 174.25, 171.12 and 164.68 (CO), 154.27, 151.12, 145.51, 133.79, and 104.31 (quat-C), 130.14, 129.59, 122.26, 121.27, 121.01, 120.78, and 119.24 (uncomplexed Ar-C), 80.21, 78.69 (Cp-C), 89.67, 77.27, 76.01, and 75.45 (complexed Ar-C), 35.34, 33.27, and 32.64 (CH_2_), 27.83 (CH_3_). ATR-FTIR; *ν_max_*/cm^−1^: 2925 (Ar-CH), 2852 (Cp-CH), 1229 (C-O-C), 2097(m), 2057(s), and 2030(s, br) (CO). Elemental analysis for C_483_H_396_O_90_Cl_12_Fe_18_P_18_F_108_Co_12_: calculated %C 46.79, and %H 3.19, and found %C 47.16, %H 4.11.

#### 3.3.12. Formation of Magnetic Materials Containing TMPs Phases

The magnetic materials containing mixture phases of TMPs was prepared using a previously described technique [30]. The dendrimer sample was weighed into a quartz boat, transferred inside a quartz tube connected to ultrapure nitrogen gas, and placed in a pyrolysis furnace. The furnace temperature was gradually raised to 900 °C over 3 h and then held constant for 4 h in a nitrogen stream. After the furnace was cooled to room temperature under a nitrogen flow (typically over a period of 3 h), the black particles were collected and weighed.

## 4. Conclusions

Magnetic materials have attracted many researchers due to their significance in several advanced applications. Here, we designed five generations of homometallic and four generations of heterometallic dendrimers, which upon pyrolysis, yielded room temperature ferromagnetic materials containing separate iron and cobalt phosphide phases. Powder-XRD, SEM and TEM, as well as SAED, revealed that the homometallic samples were a polycrystalline nature of the nearly aggregated particles and reflected single-crystalline diffraction for heterometallic magnetic ones. Dendritic effects on ferromagnetism at RT were observed with homometallic samples where *H_c_* and *M_s_* decreased with increasing precursor generation. Incorporating cobalt nanoclusters in heterometallic samples has helped to enhance their magnetization susceptibility at RT significantly compared to homometallic samples. The data gained further shows that the magnetism of the heterometallic particles was less susceptible to the influence of temperature below RT than homometallic particles, which exhibit magnetic phase transitions at temperatures around 200 K that seems to be correlated with the presence of hexagonal structures of iron/cobalt phosphides (2/1). Therefore, the dendrimer structures can be considered an important platform for the synthesis of precursors for magnetic materials containing a mixture of TMP phases, as it allows functionalization with multiple ferromagnetic metals and the control of the magnetism through dendritic effects.

## Figures and Tables

**Table 1:** Relative weight percentages, determined by the Rietveld method, between crystalline phases identified on XRD patterns for homometallic particles.

Homometallic Films	Fe_3_P	Fe_2_P	FeP	Fe	Fe_3_(PO_4_)_2_	Fe_2_(P_2_O_7_)
HOMO-G1	27.6	25.9	1.4	4.5	31.4	9.1
HOMO-G2	26.8	38.4	8.9	2.9	16.1	9.7
HOMO-G3	-	46.0	40.7	-	-	13.3
HOMO-G4	-	44.6	24.9	-	-	30.5

**Table 2:** Relative weight percentages, determined by the Rietveld method, between crystalline phases identified on XRD patterns for heterometallic particles.

Heterometallic Films	Co_2_P	Fe-FCC	Co-FCC	Co-HCP	Fe-BCC	Co_2_P-β
HETERO-G1	21.7	16.9	32.6	-	3.6	25.2
HETERO-G2	24.2	26.0	12.4	-	9.1	28.3
HETERO-G3	36.9	28.2	31.2	0.6	3.1	-
HETERO-G4	40.5	44.2	10.5	0.5	4.2	-

**Table 3:** Ferromagnetic parameters of the homometallic samples estimated from *M*(*H*) measured at RT.

Sample	Remanence *M_r_* (emu/g)	Coercitivity *H_c_* (Oe)	Saturation *M_s_* (emu/g)
HOMO-G1	1.64	175	9.7
HOMO-G2	1.07	131	9.1

**Table 4:** Ferromagnetic parameters of the heterometallic samples estimated from *M*(*H*) curves measured at RT.

Sample	Remanence *M_r_* (emu/g)	Coercitivity *H_c_* (Oe)	Saturation *M_s_* (emu/g)
HETERO-G1	1.08	73	63.4
HETERO-G2	1.32	89	60.8
HETERO-G3	1.71	81	74.0
HETERO-G4	1.19	64	75.3

## Data Availability

The data presented in this study are available in supplementary material.

## Supplementary Materials

The following are available online, Table S1: Refined lattice parameters for crystalline phases identified on XRD patterns of magnetic homometallic samples [59,60,61,62,63,64,65], Table S2: Refined lattice parameters for crystalline phases identified on XRD patterns of magnetic heterometallic samples, Table S3: Show Crystal size in Å and Crystallinity of magnetic homometallic samples, Table S4: Show Crystal size in Å and Crystallinity of magnetic heterometallic samples, Figure S1: Whole pattern profile fit for the X-ray diffraction pattern of: (a) HOMO-G1, (b) HOMO-G2, (c) HOMO-G3 and (d) HOMO-G4 by means Rietveld method: observed (red points) and calculated (black line). Positions of the Bragg reflections are depicted by green vertical bars (arranged in rows for each phase) and the observed-calculated difference is showed by blue line at the bottom, Figure S2: Whole pattern profile fit for the X-ray diffraction pattern of (a) HETER-G1, (b) HETERO-G2, (c) HETERO-G3 and (d) HETERO-G4 by means Rietveld method: observed (red points) and calculated (black line). Positions of the Bragg reflections are depicted by green vertical bars (arranged in rows for each phase) and the observed-calculated difference is showed by blue line at the bottom.

## Sample Availability

Samples of the compounds are not available from the authors.
