# Supplementary material for: Development of Ferromagnetic Materials Containing Co2P, Fe2P Phases from Organometallic Dendrimers Precursors
Source: Molecules. 2021 Nov 6;26(21):6732. doi: 10.3390/molecules26216732 (PMC8588225; doi:10.3390/molecules26216732)
Supplement: Supplementary file 1 [file molecules-26-06732-s001.zip › molecules-1416048-supplementary.pdf]

Development of Ferromagnetic Materials Containing Co_2P, Fe_2P Phases from Organometallic Dendrimers Precursors

Alaa S. Abd-El-Aziz[*,a], Maysun R. Benaaisha[a], Mohammed S. M. Abdelbaky[b], David Martinez –Blanco[c], Santiago García-Granda[b], Amani A. Abdelghani[a], Laila H. Abdel-Rahman[d], Rabin Bissessur[a],

a. *Department of Chemistry, University of Prince Edward Island, 550 University Avenue, Charlottetown, PE, C1A 4P3, Canada.*
b. *Department of Physical and Analytical Chemistry, University of Oviedo-CINN, 33006 Oviedo, Spain.*
c. *Scientific and Technical Services, University of Oviedo-CINN, 33006 Oviedo, Spain.*
d. *Chemistry Department, Faculty of Science, Sohag University, 82524 Sohag, Egypt*

[*]*Corresponding Author E-mail: abdelaziz@upei.ca*

Electronic Supporting Information

Table S1: Refined lattice parameters for crystalline phases identified on XRD patterns of magnetic homometallic samples.

Chemical Name	Formula	Space Group	*a* (Å)<br>α (º)	*b* (Å)<br>β (º)	*c* (Å)<br>γ (º)	Reference
ε-Iron Phosphide (3/1)	Fe_3P	*I* -4	9.106(8)<br>90	9.106(8)<br>90	4.460(4)<br>90	[59]
Iron Phosphide (2/1)	Fe_2P	*P* -6 2 *m*	5.866(5)<br>90	5.866(5)<br>90	3.457(3)<br>120	[60]
Iron(III) Phosphide	FeP	*P n m a*	5.191(4)<br>90	3.098(3)<br>90	5.790(5)<br>90	[61]
Iron BCC	Fe	*I m* -3 *m*	2.866(2)<br>90	2.866(2)<br>90	2.866(2)<br>90	-
Triiron Bis(phosphate(V))	$Fe_3(PO_4)_2$	*P* $2_1/c$	8.714(16)<br>90	11.175(19)<br>99.71(13)	6.248(12)<br>90	[62]
Diiron Diphosphate(V)	$Fe_2(P_2O_7)$	*P* 1	4.477(4)<br>103.52(5)	5.285(5)<br>98.32(4)	5.490(5)<br>98.37(4)	[63]

Table S2. Refined lattice parameters for crystalline phases identified on XRD patterns of magnetic heterometallic samples.

**Chemical Name**	**Formula**	**Space Group**	***a* (Å)**<br>***α* (°)**	***b* (Å)**<br>***β* (°)**	***c* (Å)**<br>***γ* (°)**	**Reference**
Cobalt Phosphide (2/1)	Co_2P	*P n m a*	5.6815(3)<br>90	3.5193(2)<br>90	6.6086(4)<br>90	[64]
Iron FCC	Fe	*F m* -3 *m*	3.5685(1)<br>90	3.5685(1)<br>90	3.5685(1)<br>90	-
Cobalt FCC	Co	*F m* -3 *m*	3.55715(8)<br>90	3.55715(8)<br>90	3.55715(8)<br>90	-
Cobalt HCP	Co	$P\ 6_3/m\ m\ c$	2.470(3)<br>90	2.470(3)<br>90	4.035(11)<br>120	-
Iron BCC	Fe	*I m* -3 *m*	2.8440(1)<br>90	2.8440(1)<br>90	2.8440(1)<br>90	-
Cobalt Phosphide (2/1) - Beta	Co_2P	*P* -6 2 *m*	5.7867(2)<br>90	5.7867(2)<br>90	3.4369(2)<br>120	[65]

Table S3: Show Crystal size in Å and Crystallinity of magnetic homometallic samples

	**Fe_3P**	**Fe_2P**	**FeP**	**Fe**	**$Fe_3(PO_4)_2$**	**$Fe_2(P_2O_7)$**	**Crystallinilty**
**HOMO_G1**	805	735		415	56	132	72.54%
**HOMO_G2**	367	370	513	319	67	132	65.54%
**HOMO_G3**	-	857	885	-	-	187	76.15%
**HOMO_G4**	-	518	621	-	-	129	80.10%

Table S4. Show Crystal size in Å and Crystallinity of magnetic heterometallic samples.

	**Co_2P**	**Fe-FCC**	**Co-FCC**	**Co-HCP**	**Fe-BCC**	**Co_2P-*β***	**Crystallinilty**
**HETERO_G1**	376	417	417	-	220	254	75.92%
**HETERO_G2**	467	417	417	-	330	628	76.51%
**HETERO_G3**	367	273	713	232	256	-	75.30%
**HETERO_G4**	536	345	598	232	437	-	86.23%

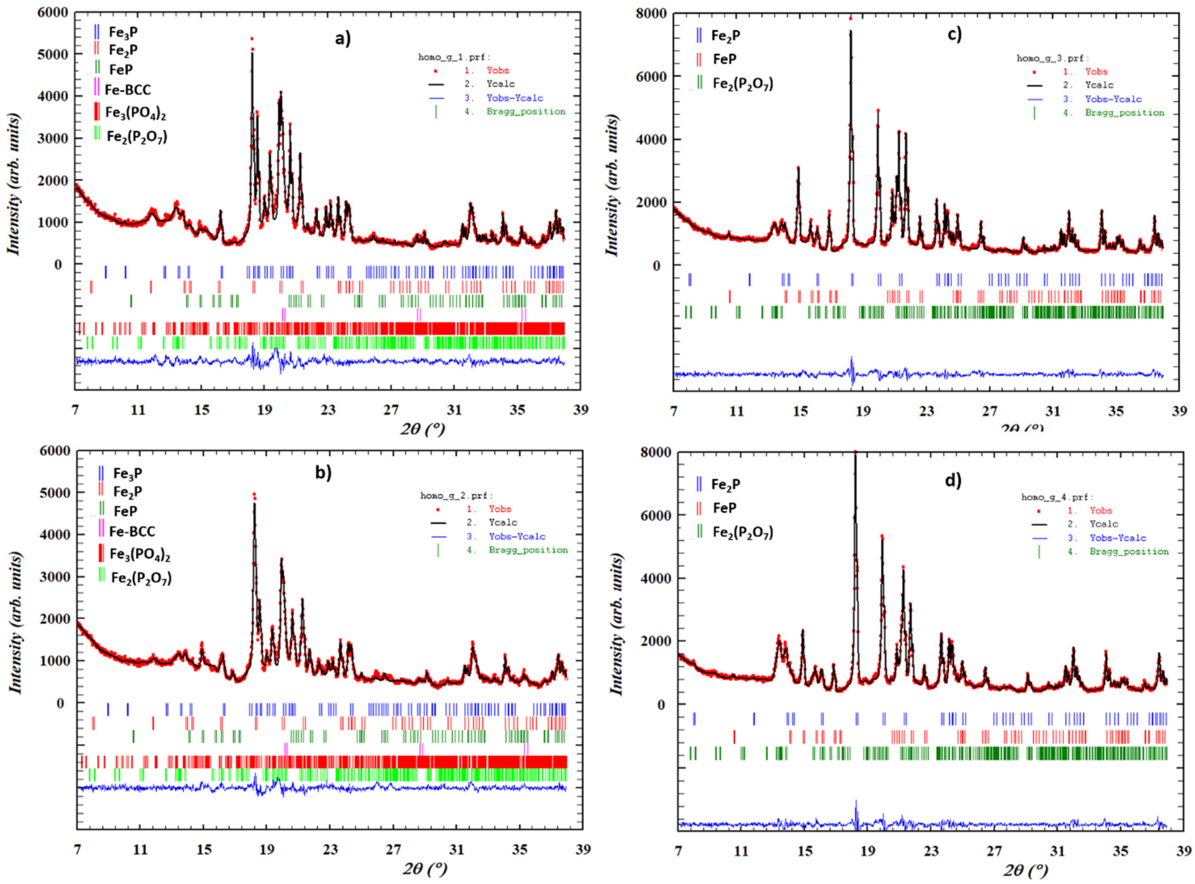

Figure S1. Whole pattern profile fit for the X-ray diffraction pattern of : a)HOMO-G1, b) HOMO-G2, c) HOMO-G3 and d) HOMO-G4 by means Rietveld method: observed (red points) and calculated (black line). Positions of the Bragg reflections are depicted by green vertical bars (arranged in rows for each phase) and the observed-calculated difference is showed by blue line at the bottom.

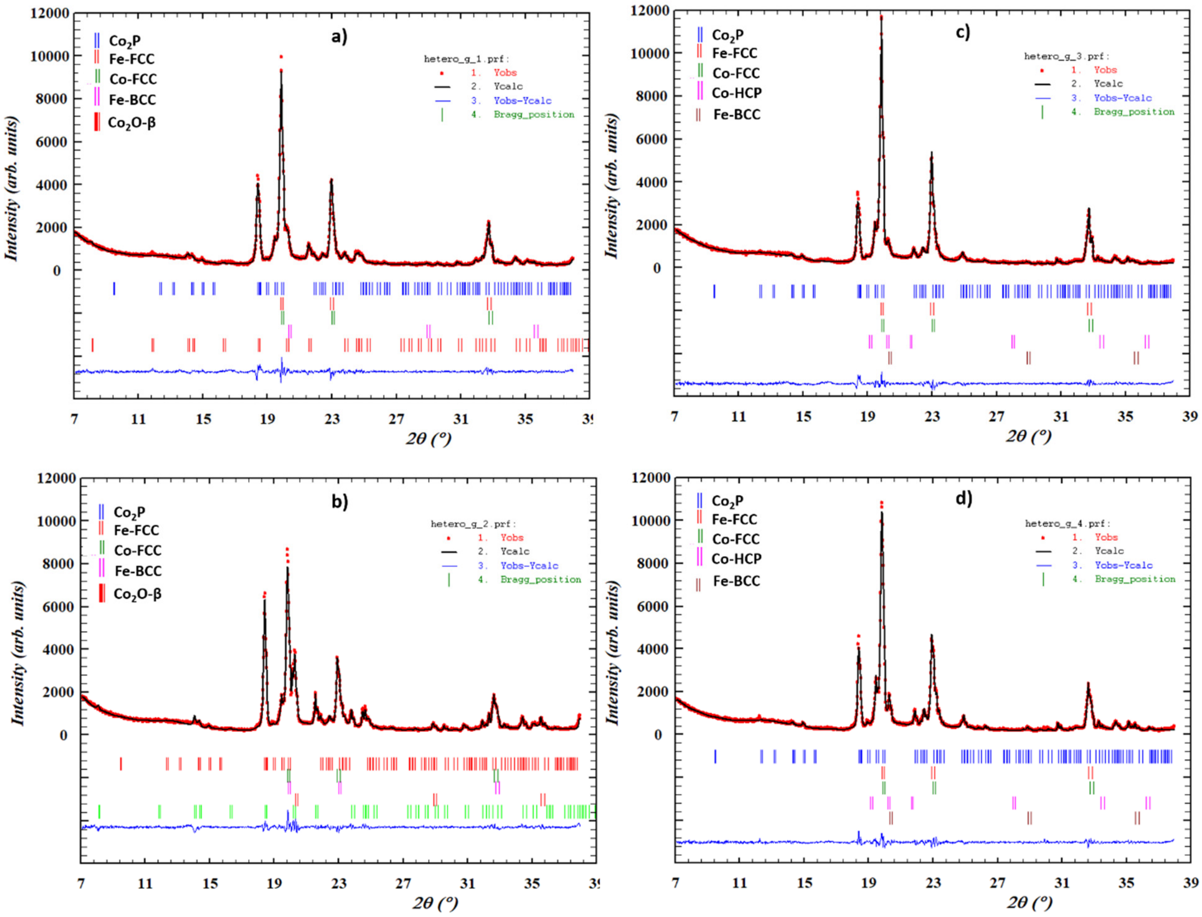

Figure S2. Whole pattern profile fit for the X-ray diffraction pattern of a)HETER-G1, b) HETERO-G2, c) HETERO-G3 and d) HETERO-G4 by means Rietveld method: observed (red points) and calculated (black line). Positions of the Bragg reflections are depicted by green vertical bars (arranged in rows for each phase) and the observed-calculated difference is showed by blue line at the bottom.
